# Blunt Abdominal Trauma With Hollow Viscus and Mesenteric Injury: A Prospective Study of 50 Cases

**DOI:** 10.7759/cureus.13321

**Published:** 2021-02-12

**Authors:** Manish Wadhwa, Rajesh Kumar, Munish Trehan, Sanjeev Singla, R Sharma, Asma Ahmed, Renuka Sharma

**Affiliations:** 1 Surgery, Maharishi Markandeshwar Medical College and Hospital, Solan, IND; 2 Surgery, Dayanand Medical College and Hospital, Ludhiana, IND; 3 General Surgery, Ramaiah Medical College and Hospital, Bangalore, IND

**Keywords:** blunt trauma, hollow viscus, jejunum, mesentery

## Abstract

Introduction

Hollow viscus injury following blunt abdominal trauma is an infrequent diagnosis. Blunt hollow viscus and mesenteric injury (HVMI) is not only an uncommon finding but its timely diagnosis is also difficult. Due to its less frequency, this injury has not been studied in detail prospectively.

Aims and objectives

The aim of this study is to determine the causes, pattern, management, and outcome of HVMI following blunt abdominal trauma.

Methodology

This study was conducted from January 2015 to June 2016 in a high-volume tertiary care trauma center and teaching hospital in North India. All patients with blunt HVMI admitted during this period were included in this study. Data were collected regarding medical history, physical findings, demographics, injury dates and times, laboratory results, diagnostic tests, delay in surgical intervention, type of surgical procedure performed, site of injury, complications, and mortality.

Results

Out of a total of 6,570 trauma admissions, 465 blunt abdominal injuries were identified, and HVMI was found only in 50 patients. The small bowel was the most common injury, with the jejunum being the most commonly involved segment. All patients were managed surgically. The mean time to operative intervention after hospital admission was 4.5 hours (IQR: 2-8 hours). Primary repair was performed in 54% of patients. Mortality rate was high in patients with HVMI (22% patients). Septic shock was the most common cause of death.

Conclusion

Hollow viscus injury in blunt abdominal trauma is not so common finding. Early diagnosis and treatment is an important but difficult task. Prognosis depends on age, associated injuries, co-morbid conditions, and delay in operative intervention.

## Introduction

Traumatic injuries are the leading cause of mortality and morbidity among patients aged 12-45 years [1]. Hollow viscus injury following blunt abdominal trauma is a not so common diagnosis [2]. Blunt hollow viscus injury accounts for less than 1% of total trauma admissions. The incidence of hollow viscus injuries following blunt abdominal trauma varies from 4% to 15% [3]. Management of blunt abdominal trauma leading to hollow viscous injury is an arduous task. Unlike blunt solid organ injuries, which are more commonly managed non-operatively, early surgical intervention is of significant importance in case of hollow viscus injury. Late diagnosis and missed injuries are important causes of increased mortality and morbidity in these patients. This study aimed to assess the cause, the pattern of gut injury, various management strategies used, and clinical outcome in patients of blunt hollow viscus and mesenteric injury (HMVI).

This article was selected for poster presentation at the Association of Surgeons of Great Britain and Ireland (ASGBI) 2020 International Surgical Congress at Glasgow in June 2020 and later on presented as e-poster during ASGBI Centenary Free Paper Series held on August 12, 2020, and the abstract was published in the British Journal of Surgery online (ASGBI edition).

## Materials and methods

This prospective study was conducted at Dayanand Medical College and Hospital (DMC&H), Ludhiana, Punjab, India, on patients of blunt abdominal trauma with HMVI from January 2015 to July 2016. Blunt abdominal trauma patients with suspected/proven HMVI diagnosed clinically/radiologically were included in this study. Patients with blunt abdominal trauma without hollow viscus injury and patients with penetrating abdominal trauma were excluded from this study. Informed consent was taken from all patients or their relatives. This study was conducted after obtaining Institutional Ethical Committee’s approval. Descriptive statistics were used to analyze the data collected in this observational study. Patients were analyzed for age, sex, cause of injury, presentation, site of injury, associated intra-abdominal and extra-abdominal injuries, delay in surgical intervention and its impact, type of surgical procedure performed, complications, need for ionotropic and ventilatory support, total hospital stay, and outcome in terms of mortality and morbidity.

## Results

Out of a total of 6,570 trauma admissions, 465 blunt abdominal injuries were identified. Of these, HMVI was found only in 50 patients. These patients with blunt HMVI were observed from the time of admission to the death or discharge from the hospital. During their hospital stay, we observed that HMVI in blunt abdominal trauma was found to be more common in the younger age group. Of the patients, 54% were in the age group of 21-40 years. In this study, blunt HMVI was found more commonly in males. Also, 45 (90%) patients were male and five (10%) patients were female, with the ratio being 9:1. Road traffic accidents were found to be the most common cause of injury. A total of 36 (72.0%) patients had sustained trauma due to roadside accidents, and the remaining patients had sustained blunt trauma from other causes such as machine injury, animal injury, handlebar injury, and fall from the height (Table 1).

**Table 1:** Distribution of the patients on the basis of cause of injury

Etiology	No. of patients	Percentage
Roadside accident	36	72.0%
Fall from height	7	14.0%
Machine injury	3	6.0%
Animal injury	2	4.0%
Cycle bar injury	2	4.0%

Patients with blunt HMVI also had associated intra- and extra-abdominal injuries. In this study, 30 (60%) patients had associated injuries. The spleen was the most commonly injured intra-abdominal organ, and skeletal system injuries were found to be the most common extra-abdominal injury (Table 2). Rectosigmoid injuries were associated with pelvic bone injuries. In six patients, HVMI was associated with only intra-abdominal solid organ injuries, 12 patients had associated extra-abdominal injuries only, and 12 patients sustained combined intra- and extra-abdominal injuries (Table 2).

**Table 2:** Associated injuries

	No. of patients
Spleen	9
Liver	7
Pancreas	2
Renal	3
Diaphragm	2
Skeletal system	22
Blunt trauma chest	7
Head injury	1
Faciomaxillary injuries	1

In this study, the median delay between injury and arrival at the hospital for these patients was 12 hours (interquartile range [IQR]: 6-24 hours). The median delay between presentation at hospital and operative intervention was 4.5 hours (IQR: 2-8 hours). Initial management of major trauma patients by non-specialist doctors working at low-volume under-equipped centers is still very common in India. In our study also, patients who presented late at our hospital, a tertiary trauma care facility, were initially managed by general practitioners or by specialists at less equipped centers, and, in some cases, patients took analgesics from local chemist shops to control abdominal pain. Time consumed between presentation at the Emergency Department and shifting to theatre for surgery was because of the time spent in initial resuscitation and diagnostic workup in the hospital.

In this study, three patients sustained gastric injuries. All three patients had associated splenic injuries, and pancreatic injury was seen in one of these three patients. All three patients were taken to the operation theater for splenectomy, and gastric injury was found during surgery. The small bowel was found to be the most common site to get injured in 22 (44%) patients followed by mesentery in 12 (24%) patients. Only three patients had a combined mesenteric and bowel injury. The jejunum was injured in 12 patients with small bowel injury (50%). The blunt duodenal injury was found in two cases, both secondary to handlebar injury. Both cases had a gradual onset of symptoms. The pancreatic injury was found to be associated with one patient. Both patients had superficial wound dehiscence during the postoperative hospital course. The Ileum bore the brunt in the remaining eight patients with small bowel injury. Ten (20%) patients sustained large bowel injury. Sigmoid colon, rectum, and cecal injuries were seen in five, three, and two patients, respectively (Table 3).

**Table 3:** Site of injury

	Site	No. of patients	Percentage
Stomach (6%)	Stomach	3	6.0%
Small intestine (44%)	Duodenum	2	4.0%
	Jejunum	12	24.0%
	Ileum	8	16.0%
Large bowel (20%)	Sigmoid colon	5	10.0%
	Rectum	3	6.0%
	Caecum	2	4.0%
Mesentery (24%)	Jejunum mesentery	4	8.0%
	Ileum mesentery	5	10.0%
	Mesosigmoid	3	6.0%
Combined (6%)	Mesentery and small bowel Injury	3	6.0%

All patients underwent an X-ray of the chest; however, only 10 (20%) patients were found to have pneumoperitoneum. Nine (18%) patients with hemodynamic instability, signs of peritonitis, and free fluid on ultrasonography (USG) were taken up for surgery without them undergoing a CT scan. Also, 31 (62%) patients with stable hemodynamic status were taken up for surgery based on their abdominal contrast-enhanced CT (CECT) findings. In our study, in 10 (20%) patients, HMVI was missed on initial clinical examination and radiological investigations including CT scan. Among these, there were two mesenteric injuries, two small bowel injuries, and six large bowel injuries. Lawson et al. also observed that in patients with blunt abdominal trauma, bowel injuries were most commonly missed; therefore, they concluded that a high index of suspicion and tertiary survey are required for timely diagnosis and treatment [4].

Regarding treatment, all of the patients were managed surgically. Primary repair of the perforation was the most commonly used technique in 27 (54%) patients, resection and anastomoses was carried out in 19 (38%) patients, and Hartmann's procedure was performed in 4 (8%) patients. Diversion ileostomy was performed in 34% of patients. In this study, all of the three gastric injuries were managed by primary repair of serosal tears. The duodenal injury was found in two patients in this study. One patient was managed with primary repair and feeding jejunostomy. The second patient was first managed with primary repair and peritoneal drainage at some other hospital but developed a biliary leak from the repair site and later on managed by closure of the perforation site along with pyloric exclusion and gastrojejunostomy at DMC&H. In our study, 58.3% (7/12) patients with mesenteric injury had gangrenous changes in the bowel, which were managed by resection and anastomosis (Table 4).

**Table 4:** Type of surgical procedure *All three patients had serosal tears repaired primarily. **The second patient was managed by primary closure with pyloric exclusion and gastrojejunostomy.

	Primary closure without diversion	Primary closure with diversion	Resection anastomosis without diversion	Resection anastomosis with diversion	Hartmann’s procedure
Stomach (3 patients)	3/3 patients* (100%)	–	–	–	–
Duodenum (2 patients)	1/2 patients (50%)	1/2 patients** (50%)	–	–	–
Jejunum (12 patients)	6/12 patients (50%)		6/12 patients (50%)		
Ileum (8 patients)	1/8 patients (12.5%)	4/8 patients (50%)	1/8 patients (12.5%)	2/8 patients (25%)	–
Caecum (2 patients)	2/2 patients (100%)	–	–	–	–
Colon (5 patients)	–	1/5 patients (20%)	–	–	4/5 patients (40%)
Rectum (3 patients)	–	3/3 patients (100%)	–	–	–
Mesentery (12 patients)	5/12 patients (41.67%)		3/12 patients (25%)	4/12 patients (33.33%)	–
Combined mesentery with small bowel (3 patients)	–	–	–	3/3 patients (100%)	–

In this study, the most common complication was surgical site infection in 13 (26%) patients(Table 5).

**Table 5:** Complications

Type of complication	No. of patients	Percentage
Surgical site infection	13	26.0%
Wound dehiscence	10	20.0%
Stump leak	2	4.0%
Anastomotic leak	1	2.0%
Enterocutaneous fistula	2	4.0%
Stoma related	1	2.0%
Chest complications	13	26.0%
Cardiac complications	3	6.0%
Paralytic ileus (prolonged)	1	2.0%
Acute renal failure	2	4.0%
Decompensated liver disease	2	4.0%
Septic shock	9	18.0%

Ionotropic support was required in 52.0% (26) patients. Ventilatory support was required in 58.0% (28) patients. Mean packed cell (PC), fresh frozen plasma (FFP), and random donor platelet (RDP) usage was 2.94 units, 2.68 units, and 0.40 units, respectively. Admission to intensive care unit (ICU), care under intensivist, and blood product usage was required in those patients who had sustained associated solid viscus or significant skeletal injuries.

The mortality rate in our study was 22.0% (11 patients). Septicemic shock with multi-organ failure was the most common cause of death in our study. Out of 11 deaths. Eight were due to septic shock with multi-organ failure and three due to worsening of their preexisting medical conditions. Six patients were older than 50 years. Seven patients had surgical intervention after 24 hours. Nine patients had associated injuries, four patients had only associated injuries, and five patients had associated injuries as well as co-morbid medical conditions that worsened during the hospital stay (Table 6).

**Table 6:** Mortality MI, myocardial infarction

	No. of patients	Percentage
Septic shock with multiorgan failure	8	16.0%
MI/cardiac causes	1	2.0%
Decompensated liver disease	2	4.0%

Hospital stay in these patients depended on various factors including the severity of the injury, associated injuries, delay in surgical intervention, co-morbid medical conditions, and complications. The mean duration of total hospital stay in our patients was 19.02 days. The mean duration of the ward and ICU stay was 12.27 days and 9.07 days, respectively.

## Discussion

In this study, we managed 50 patients of blunt abdominal trauma with HMVI from January 2015 to July 2016. Out of the total trauma patients admitted during this period in DMC&H, 465 patients sustained blunt abdominal trauma and only 50 (10.75%) of these blunt abdominal trauma patients were diagnosed with HMVI, which is similar to incidence found in other studies. Patients' age varied from 4 to 80 years. The highest incidence was found among patients in the age group of 21-40 years. Ayoade et al. also found this age group to have the highest incidence (68.90%) in their study [5]. This might be for the reason that this age group forms a major segment of the workforce.

The majority of these patients sustained injuries due to roadside accidents. Other causes such as falls from height, machine injury, animal injury, and cycle bar injury (both in children) have also been implicated in HVMI.

The length of hospital stay has been reported to be an important measure of morbidity among trauma patients. Hospital stays varied from day 1 to day 44. The mean duration of the total hospital stay was 19.02 days. Short stay was there only in patients who had early death. In our study, one patient died on the first postoperative day and one patient died on the second postoperative day. In all other patients, hospital stay gets prolonged due to delayed presentation, hemodynamic instability at the time of presentation, associated co-morbidities, and postoperative complications.

In our study, 17 (34%) patients had associated co-morbidities (e.g., diabetes mellitus in eight [16%] patients). Gad et al. also found co-morbidities in 19.4% patients [6]. Abdominal trauma is commonly associated with other injuries that complicate the management and also affect the outcome [7]. In our study, 30 (60%) patients had associated intra-abdominal and extra-abdominal injuries. The most common solid organ injured was the spleen (nine patients) followed by the liver (seven patients). A study by Currie et al. also showed similar results [8]. A total of 24 (48%) patients had associated extra-abdominal injury. The skeletal injury was the most common extra-abdominal injury followed by a blunt chest trauma. Among skeletal injuries, pelvic injuries were seen in 37.5% of patients. This is comparable to a study conducted by Ayoade et al., which showed skeletal injuries to be the most common associated extra-abdominal injury and pelvic fractures to be the most common among skeletal injuries. In our study, patients with associated injuries were found to have prolonged hospital stay and high rates of postoperative complications, leading to the increased total cost of treatment, which is in agreement with other studies. Other than co-morbidities and associated injuries, delay in diagnosis and surgical intervention also affect the final outcome. In this study, patients operated after 24 hours of injury also had prolonged hospital stay, as well as increased need for ionotropic support, ventilatory support, and blood/blood product transfusion. These patients also had increased incidence of postoperative wound and chest-related complications.

Diagnosis of bowel and mesentery injury was made based on signs of peritonism, patient's hemodynamic status, and radiological findings. Radiological tests used to evaluate patients with blunt trauma include X-ray of the chest, USG of the abdomen, and CECT of the abdomen. Ultrasonography is an easily accessible, less expensive, and non-invasive investigation. The presence of free fluid without solid organ injury is considered to be suggestive of hollow viscus and/or mesenteric injury. CT findings that were considered diagnostic for bowel injury were contrast extravasation and/or extraluminal air. Findings that were non-diagnostic but suggestive were free fluid without solid organ injury with small bowel thickening and dilatation [7]. Killeen et al. found a high level of accuracy for detection of bowel injuries, with an accuracy of 86%, sensitivity of 94%, and a positive predictive value of 92% for CT of the abdomen [9]. CT of the abdomen is the most sensitive and specific diagnostic tool to document abdominal injury; however, its diagnostic value for patients with colon injury remains controversial. As the incidence of colon injury due to blunt abdominal trauma is low, the lack of a definitive diagnostic method for the same can lead to delays in diagnosis and treatment. Presently, there is no single method to accurately diagnose colon injuries caused by blunt trauma. Some studies show the efficacy of physical examination and in-hospital observation for the diagnosis of colonic injuries, but the absence of findings on examination does not rule out the colonic injury. In our study, we missed gut injury in 10 patients, and later on these patients were diagnosed on repeat CT scan of the abdomen due to deterioration of their clinical status.

In this study, the small bowel was the most common site to get injured, which was the same as reported by other studies [10-13]. The jejunum was injured in 12 (50%) patients with small bowel injury. Fakhry et al. also showed that most blunt abdominal injuries affect the jejunum [12]. Small bowel injuries mainly happen within proximity to the duodenojejunal flexure and ileocecal junction, i.e., close to fixed points, and a majority of them are located on the anti-mesenteric border. Large bowel injuries generally occur in penetrating trauma, whereas in blunt trauma, it is a less common finding. In our study, the large bowel was injured less frequently in comparison to small bowel injury. This has also been reported in various other studies that colonic injuries occurred less frequently than small bowel injuries [14]. Sule et al. also found colonic injuries to be less frequent than small bowel injury in their study. This is mainly due to its location and the lack of redundancy, which prevents the formation of closed loops [14,15]. The mesentery was the second most common organ to get injured. Mesenteric injury varied in severity from simple contusions to complete transection, leading to bowel ischemia and gangrene. Clinical manifestations of patients with isolated mesenteric vascular injury include features of intra-abdominal bleeding leading to hemodynamic instability and peritonism. Delayed manifestations of mesenteric injuries might be due to sepsis, bowel ischemia, and gangrene. In our study, 5/12 patients with mesenteric injury had mesenteric vascular injury and presented with hemodynamic instability, and 7/12 had ischemic and gangrenous bowel and presented with peritonitis.

Regarding treatment, all patients were managed surgically. Primary repair of the perforation was the most commonly used technique. Diversion ileostomy was performed in those patients (with ileal injury/colonic injury/cecal/rectal/mesenteric injury) who were hemodynamically unstable, those who had multiple co-morbidities, and moribund patients with the grossly contaminated peritoneal cavity. Resection and anastomosis was performed when there were multiple perforations in proximity or when bowel viability was compromised, as found in some patients with mesenteric injuries. Cecal injuries were repaired primarily. Hartmann’s procedure was preferred for sigmoid colon injuries, and rectal injuries were repaired primarily with diversion. Hartmann’s procedure was performed in four (80%) patients of sigmoid injury, and one (20%) patient was subjected to primary repair with diversion. All patients (three) with rectal injuries were repaired primarily with proximal diversion. In our study, 75% of sigmoid colon injuries were diagnosed late, which led to complicated postoperative hospital course [16]. Fakhry et al. also had complicated postoperative course in patients with late diagnosed colonic injuries [12].

The most common complication was surgical site infection in 13 (26%) patients. This could be due to contamination of peritoneal cavity by bowel contents, which further led to surgical site sepsis. Surgical site infections were managed with aseptic dressings and intravenous antibiotics as per culture and sensitivity reports. Wound dehiscence was one of the most dreadful complications, which led to an increased hospital stay. In our study, 10 (20%) patients had wound dehiscence. Patients with wound dehiscence were managed by secondary suturing and daily dressings. In this study, two (4%) patients had enterocutaneous fistula (low output) development in the postoperative period. Both these patients with enterocutaneous fistula were managed conservatively without any intervention. One patient had an anastomotic leak, which was managed surgically. Two patients had rectal stump leaks, which were managed by percutaneous drain insertion.

Mortality rates quoted from blunt intestinal trauma range from 10% to 30% [17]. The mortality rate in our study was 22.0%. The high mortality rate in our study might be because of the high rate of associated injuries. Reports have shown that mortality increases with the number of associated injuries [18]. Other than associated injuries, co-morbid medical conditions also adversely affect the management outcome.

## Conclusions

Blunt HMVI is an uncommon phenomenon with a high mortality rate. Prognosis depends on early diagnosis and surgical intervention, associated injuries, and other co-morbid medical conditions. Associated injuries play a significant role in the survival. The diagnosis of blunt bowel injury is a daunting task. Repeated examinations, greater vigilance, and tertiary survey have considerable importance for timely diagnosis.
